# Mental health trajectories of adolescents treated with psychotropic medications: insights from the european milestone study

**DOI:** 10.1038/s41380-025-03307-3

**Published:** 2025-10-17

**Authors:** Marta Magno, Donato Martella, Silvia Leone, Giovanni Allibrio, Angelo Bertani, Elisa Caselani, Patrizia Conti, Samuele Cortese, Gwen Dieleman, Tomislav Franic, Suzanne Gerritsen, Deborah Maffezzoni, Francesco Margari, Ottaviano Martinelli, Fiona McNicholas, Rocco Micciolo, Renata Nacinovich, Diane Purper Ouakil, Adriana Pastore, Francesco Rinaldi, Paramala Santosh, Paolo Scocco, Ulrike Schulze, Swaran Singh, Antonella Squarcia, Paolo Stagi, Cathy Street, Elena Toffol, Helena Tuomainen, Larissa S. van Bodegom, Stefano Vicari, Giovanni de Girolamo

**Affiliations:** 1 Unit of Epidemiological and Evaluation Psychiatry, IRCCS Istituto Centro San Giovanni di Dio Fatebenefratelli, Brescia, Italy; 2 Child and Adolescent Neuropsychiatry, ASST Bergamo Ovest, Treviglio, Italy; 3 Unit of Youth Psychopathology, ASST Santi Paolo e Carlo, Milan, Italy; 4 Child and Adolescent Neuropsychiatry, ASST Lariana, Como, Italy; 5 Centre for Innovation in Mental Health, School of Psychology, Faculty of Environmental and Life Sciences, University of Southampton, Southampton, UK; 6 Clinical and Experimental Sciences [CNS and Psychiatry], Faculty of Medicine, University of Southampton, Southampton, UK; 7 Solent NHS Trust, Southampton, UK; 8 Hassenfeld Children’s Hospital at NYU Langone, New York University Child Study Center, New York City, New York USA; 9 DiMePRe-J-Department of Precision and Rigenerative Medicine-Jonic Area, University of Bari “Aldo Moro”, Bari, Italy; 10 Department of Child and Adolescent Psychiatry and Psychology, Erasmus Medical Center, Rotterdam, The Netherlands; 11 University Hospital of Split, Department of Psychiatry, Medical School University of Split, Split, Croatia; 12 Dept of Basic Medical Sciences, Neuroscience and Sensory Organs, University of Bari, Bari, Italy; 13 Child and Adolescent Neuropsychiatry, ASST Lecco, Lecco, Italy; 14 Lucena Clinic, Child and Adolescent Mental Health Services, Saint John of God, Dublin, Ireland; 15 School of Medicine and Medical Science, University College Dublin, Belfield, Dublin 4, Dublin, Ireland; 16 Children’s Health Ireland at Crumlin, Dublin, Ireland; 17 Centre for Medical Sciences, Department of Psychology and Cognitive Sciences, University of Trento, Trento, Italy; 18 School of Medicine and Surgery, University of Milano-Bicocca, Milan, Italy; 19 Child and Adolescent Neuropsychiatry, IRCCS San Gerardo dei Tintori, Monza, Italy; 20 University Hospital of Montpellier, MPEA1 Montpellier, France; 21 INSERM U 1018 CESP, Villejuif, France; 22 Policlinico Hospital, Unit of Adolescent Psychiatric Emergency, Bari, Italy; 23 Child and Adolescent Neuropsychiatry, ASST Valcamonica, Breno, Italy; 24 Institute of Psychiatry, Psychology and Neurosciences, King’s College London, and the Maudsley Hospital, London, UK; 25 SOPROXI Onlus, Padova, Italy; 26 University of Ulm, Department of Child and Adolescent Psychiatry/ Psychotherapy, Ulm, Germany; 27 Centre for Psychiatry Calw, Clinic for Child and Adolescent Psychiatry and Psychotherapy, Böblingen, Germany; 28 Warwick Medical School, University of Warwick, Warwick, United Kingdom; 29 Child and Adolescent Neuropsychiatry, Department of Mental Health, AUSL di Parma, Parma, Italy; 30 Child and Adolescent Neuropsychiatry, Azienda USL Toscana Centro, Firenze, Italy; 31 Adolescent Neuropsychiatry Unit, Bambino Gesù Children’s Hospital, Rome, 00165 Italy; 32 Warwick Clinical Trials Unit, Warwick Medical School, University of Warwick, Coventry, UK; 33 Warwick Medical School, University of Warwick, Coventry, UK; 34 Coventry and Warwickshire Partnership NHS Trust, Coventry, UK; 35 Yulius Mental Health Organization, Yulius Academy, Dordrecht, Netherlands; 36 Department of Child and Adolescent Psychiatry and Psychology, Erasmus Medical Center, Rotterdam, Netherlands; 37 ARQ National Psychotrauma Centre, Diemen, Netherlands; 38 University Hospital Split, Split, Croatia; 39 School of Medicine, University of Split, Split, Croatia; 40 Centre Hospitalier Universitaire de Montpellier, Saint Eloi Hospital, Montpellier, France; 41 Josefinum Augsburg, Klinik für Kinder- und Jugendpsychiatrie und -Psychotherapie, Augsburg, Germany; 42 NIHR Mental Health Policy Research Unit, Division of Psychiatry, University College London, London, UK; 43 Department of Child & Adolescent Psychiatry, Institute of Psychiatry, Psychology and Neuroscience, King’s College London, London, UK; 44 Centre for Interventional Paediatric Psychopharmacology and Rare Diseases, South London and Maudsley NHS Foundation Trust, London, UK; 45 HealthTracker, Kent, UK; 46 Department of Neurosciences, Centre for Clinical Psychiatry, KU Leuven, Leuven, Belgium; 47 Child and Adolescent Psychiatry Department, Fontan Hospital, CHU Lille, Lille, France; 48 Department of Child and Adolescent Psychiatry and Psychotherapy, University of Ulm, Ulm, Germany; 49 Rees Centre, Department of Education, University of Oxford, Oxford, UK

**Keywords:** Depression, ADHD, Bipolar disorder, Schizophrenia, Autism spectrum disorders

## Abstract

**Abstract:**

The transition from Child and Adolescent (CAMHS) to Adult Mental Health Services (AMHS) can be challenging. Drawing on the sample of the European MILESTONE project, we explored changes in clinical profiles and treatment outcomes in adolescents transitioning to AMHS over two years, focusing on different pharmacological treatment patterns. The sample (N = 690; mean age: 17.7 years; SD = 0.29) was categorised into three groups based on medication patterns: *continuous* (Group 1), *intermittent* (Group 2), and *never medicated* (Group 3). Participants underwent four evaluations over two years using tools measuring psychopathology and functioning, including the Health of the Nation Outcome Scale for Child and Adolescents (HoNOSCA) and ASEBA Battery. We employed repeated-measures models to analyse clinical rating changes and a two-way mixed ANOVA to assess interaction between time and groups. Group 3 had significantly lower mean HoNOSCA ratings than Groups 1 and 2 (p < 0.001), indicating better mental health. By the last time point (T4), the factors associated with a reduced risk of severe illness included an improvement in the risk of suicide attempts (p = 0.038), enhanced everyday functional skills (p = 0.008), higher quality of life (p = 0.001), and being male (p = 0.020). The ASEBA Battery showed Group 1 had more internalising symptoms, while Group 2 had more externalising symptoms than Group 3. During the transition from CAMHS to AMHS, continuous medication was associated with higher symptom severity than intermittent or no pharmacological treatment. This may reflect either a more severe initial symptomatology requiring sustained pharmacotherapy or a medication-related paradox, whereby symptoms persist or intensify owing to treatment resistance or side effects.

**Trial registration:**

“MILESTONE study” registration: ISRCTN ISRCTN83240263 Registered 23 July 2015; ClinicalTrials.gov NCT03013595 Registered 6 January 2017.

## Background

Adolescence is a period of increased susceptibility to mental health challenges owing to significant physical, emotional, and social changes [1]. Hormonal fluctuations, ongoing brain maturation [2], and external stressors such as academic demands and the search for social identity influence this vulnerability [3, 4]. In addition, individual traits, family dynamics, and social interactions can affect the mental well-being of young people. Factors such as low self-esteem, challenging family circumstances, and peer victimisation may lead to depression, anxiety, and behavioural problems [5]. A meta-analysis showed a rising prevalence of mental disorders among young people in Europe; almost one in five young people in Europe was found to suffer from mental disorders, with a pooled prevalence rate of 15.5% in 14 European countries [6]. Other epidemiological investigations have indicated that approximately 14% of individuals aged 10-19 experience such conditions during this phase of life [7–10], which can significantly affect functioning in later life [11]. Although the median age of onset for many disorders in males and females is similar, there are significant sex differences in the lifetime prevalence of mental disorders [12]. Anxiety and major depressive disorders are more commonly experienced in female population, while impulse control and substance use disorders are more prevalent in male population. [13].

In recent decades there has been a corresponding global increase in the prescription of psychotropic medications for children and adolescents [14], but changes in physical development during childhood and adolescence may contribute to suboptimal treatment effectiveness and tolerability in young individuals. This may lead to unexpected outcomes in the young population [15]. For young people facing transition from Child and Adolescent Mental Health Services (CAMHS) to Adult Mental Health Services (AMHS), ensuring seamless continuity of care and pharmacological treatment for those in need is important but can be challenging. This phase occurs during a vulnerable period and is compounded by existing mental health challenges [16, 17]. Adolescents taking psychotropic medications may require treatment adjustments during this transition, whereas those not taking such medications tend to have higher dropout rates [18]. Close collaboration between CAMHS and AMHS professionals is essential to ensure continuity of care and address changing medication needs and/or potential side effects. Ineffective transition management can lead to care disruptions, treatment gaps, and an increased risk of relapse or mental health deterioration.

To explore and address the treatment gaps that occur during transition, we conducted a cluster randomised controlled trial (cRCT) of Managed Transition with a parallel longitudinal cohort study (together called the “MILESTONE study”) as part of the wider the MILESTONE project (2014–2019) [19]. The aim of the project was to improve the understanding and strengthen transitional care processes from CAMHS to AMHS across European healthcare systems. This study was a secondary analysis of the longitudinal MILESTONE study data which aimed to explore clinical profile changes in adolescents transitioning from CAMHS to AMHS over a two-year follow-up. The aim of this study was to examine mental health trajectories and treatment outcomes in young people with different medication regimes. We achieved this by examining how sociodemographic factors interact with psychopathological severity beyond single diagnostic categories and using a comprehensive evaluation method integrating patient self-reports and clinician-rated reports.

## Method

### Study design and participants

The MILESTONE study was designed to investigate service utilisation, mental health, and additional outcomes during a 2-year monitoring period among a group of 1004 young individuals aged 17-19 who have reached the upper age limit of their CAMHS across eight European countries.

**Fig. 1:**
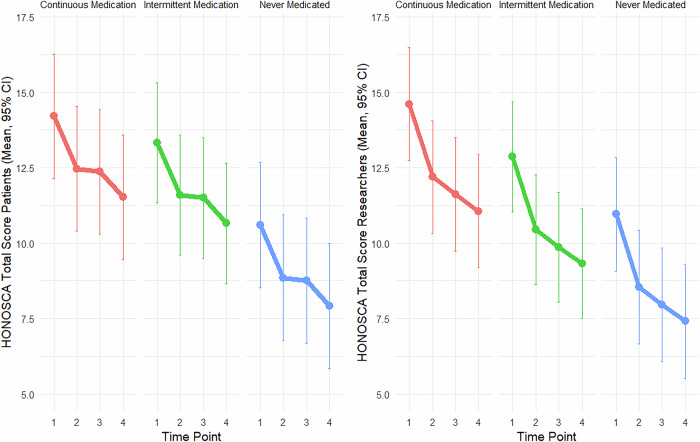
Honosca total ratings: patients’ vs. clinicians’ ratings by medication status.

Previous studies have described their study design and recruitment procedures in detail [19]. In the present study, we focused on analysing data from all participants (N = 1004). Participants who did not report any information regarding psychopharmacological treatment were excluded from this study (Fig. 1S): finally, data from 690 individuals, including both males and females, were analysed. Participants were eligible if they met the following criteria: (i) they were CAMHS users and were within one year of the upper age limit (almost 18 years old), or were up to three months older (mean = 17.68; SD = 0.29) if they were still under CAMHS care during the study period**;** (ii) they had a documented psychiatric diagnosis or regularly attended CAMHS; (iii) they had an IQ of more than 70 or showed no evidence of intellectual impairment, as determined by clinician assessment or previous diagnosis, and (iv) were able to complete all required assessments. The sample size was estimated for three groups assessed at four time points over a 24-month period. This estimation focused on the change from baseline to 24 months across three clinical scales: HoNOSCA, ASEBA Internalizing, and ASEBA Externalizing, which were identified as the primary outcomes.

Assuming a within-subject correlation of approximately 0.6, with baseline standard deviations ranging from 8–10 points and a small to moderate group effect (Cohen’s f ≈ 0.12), the required sample size was calculated using an ANOVA framework based on change scores. The goal was to achieve 80% power with a significance level of α = 0.05.

After applying Bonferroni correction for the three endpoints and accounting for an anticipated 20% attrition rate, the final required sample size was approximately 153 participants per group.

### Measures, procedures, and variables

Participants took part in four assessments over a 24-month follow-up period: 3 months before transitioning from CAMHS to AHMS (T1), 9 months after T1 (T2), 15 months after T1 (T3), and 24 months after T1 (T4). At T1, all patients received CAMHS treatment. Following obteined informed consent, participants and parents took part in a baseline assessment (T1) in CAMHS approximately six months before reaching the upper age limit. Trained researchers gathered sociodemographic data and assessed care needs via interviews conducted at CAMHS, at home, or by telephone, accommodating the participants’ preferences. Sociodemographic information was sourced from clinicians, parents, medical files, and online questionnaires were accessed via HealthTracker™ (https://www.healthtracker.co.uk).

The assessment battery administered included (see Table 2S): (1) HoNOSCA (Range 0-52) [20, 21], used to assess the need for care based on a wide range of problems (behaviour, impairment, symptoms, and social functioning); it was filled through semi-structured interviews with the young person, and (where possible) with parent/caregiver and the relevant clinician (or review of medical records if the relevant clinician was unavailable); (2) ASEBA battery [22, 23], including Youth Self Report (YSR) or Adult Self Report (ASR), and the parent/caregiver-reported Child Behaviour Checklist (CBCL) or Adult Behaviour Checklist (ABCL) to assess dimensions of emotional and behavioural problems; (3) Specific Levels of Functioning Scale (SLOF) (Range 43–125) [24, 25], a parent-reported measure used to assess levels of functioning of the young person from the parent/caregiver’s perspective (4) Life events questionnaire (Range 0-13), an ad hoc tool specifically developed within the MILESTONE project, administered to patients and used to assess significant life events, such as accidents, deaths in the family, separation and job loss of the parent/caregiver; (5) World Health Organization Quality of Life Brief (WHOQOL-Bref) (Range 0–100) [26], administered to patients to assess quality of life and covering physical and psychological health, social relationships, and the current environment of the young patients; (6) Bullying, adapted from Retrospective Bullying and Friendship Interview Schedule, a patient-completed questionnaire used to assess the experiences with bullying in different settings (e.g., at school, home, and college) [27]; (7) Clinical Global Impression - Severity scale (CGI-S) (Range 1-7) rated by the clinician and used to assess the severity of the patient’s illness at the time of assessment [28]. Information regarding psychopharmacological treatment were collected using from the clinician, from clinical documentation and directly from patients using a form created ad hoc: whenever there was a disagreement between these three sources of information, we retained the information provided by the clinician.

To assess the link between psychotropic prescriptions and patients’ clinical course, we categorised our sample into three groups: (i) *‘continuous medication*,’ comprising patients prescribed at least one psychotropic medication across all four time points; (ii) *‘intermittent medication*,’ including patients prescribed at least one psychotropic medication at any time points; and (iii) *‘never medicated*,’ consisting of patients never prescribed psychotropic medications throughout the four time points.

### Statistical analysis

Continuous variables were compared between the three psychotropic medication groups using t-tests, while categorical variables were assessed using the χ^2^ test. As mentioned previously, we selected patients from the entire sample (N = 1004) with available psychotropic medication data (including those who declared that they weren’t receiving medications) at all four time points (N = 690). See Table 1S for the characteristics of the excluded sample. To address missing clinical scores (HoNOSCA and ASEBA), we used multiple imputations [29] across all four time points to identify temporal patterns before fitting the mixed models. We used logistic regression analysis with a binomial family at the final time point to assess the association between sociodemographic variables and the clinical course/outcomes across the three psychotropic medication groups. The CGI-S, which assesses the severity of symptoms, dichotomised patients into no/mild severity (0-1-2-3) and moderate/severe severity (4-5-6-7) groups. Patients who had experienced bullying at least once were classified as victims; if they have experienced both, bullying and being a victim, they were labelled as bullies. General health and social functioning over the four time points were evaluated using HoNOSCA total ratings, while emotional and behavioural problems were assessed using ASEBA battery ratings from patients and parents. First, we employed repeated-measures models with to assess the interaction effect between time and psychotropic medication groups, accounting for the random effects of each subject and of each country. Finally, we conducted a two-way ANOVA to explore the interaction between time point and the type of assessment. Analyses were conducted using R software version 4.3.2 with a significance threshold of 0.05. The code used for the analyses is available from the corresponding author upon reasonable request.

## Results

### Sociodemographic and clinical characteristics of participants

Table 1 shows the sociodemographic characteristics of the 690 adolescent patients from the 9 centres in eight European countries. The sample had a mean age of 17.7 years (SD = 0.29), with over 80.0% Caucasians and 64.1% women. In terms of household composition, 55.9% of adolescents lived with their biological parents, 7.5% lived with a single parent, and only 33.3% had other living arrangements (including foster care or residential facilities). Almost 70% of the participants did not attend school or university at the time of recruitment, whereas 5.8% were currently enrolled in school.

**Table 1:** Sociodemographic characteristics of young people in the milestone cohort assessed at baseline (T1, N = 690).

	N (%) or mean (SD)
**Gender**	
*Female*	442 (64.1%)
*Male*	248 (35.9%)
**Age**	17.68 (0.29)
**Ethnicity**	
*Caucasian*	552 (80.0%)
*Other ethnic groups*	55 (7.9%)
*Missing*	83 (12.1%)
**Country**	
*Belgium*	79 (11.5%)
*Croatia*	43 (6.2%)
*France*	59 (8.6%)
*Germany*	57 (8.3%)
*Ireland*	21 (3.0%)
*Italy*	171 (24.8%)
*Netherlands*	117 (16.9%)
*UK London*	37 (5.4%)
*UK West Mids*	93 (13.5%)
*Missing*	13 (1.8%)
**Living situation**	
*With biological parents*	386 (55.9%)
*With one biological parent*	52 (7.5%)
*Adoptive/foster parent(s) or other living arrangements*	230 (33.3%)
*Missing*	22 (3.3%)
**Current education**	
*Secondary/vocational*	12 (1.7%)
*Higher (under/postgraduate)*	28 (4.1%)
*No current school attendance*	495 (71.7%)
*Other*	26 (3.8%)
*Missing*	129 (18.7%)

### Sociodemographic and clinical associations among the three psychotropic medication groups

Table 2 shows the clinical characteristics of the patients. Across all three groups, most participants were rated as “borderline/mildly/moderately ill” on the CGI-S (58.1% in Group 1, 58.9% in Group 2, and 62.5% in Group 3), with a smaller proportion rated as “not at all ill” (8.1% in Group 1, 5.9% in Group 2, and 14.5% in Group 3). There was significant difference between the groups (p = 0.018). Group 3 had a significantly lower mean HoNOSCA score (9.3) than Groups 1 (13.9) and 2 (12.8) (p < 0.001), indicating better mental health. Group 3 also had a lower percentage (17.0%) of participants with a lifetime history of suicide attempts than Groups 1 (30.1%) and 2 (29.6%). Significant differences (p < 0.001) were observed between these groups in the proportion of participants with one or more suicide attempts. Concerning non-accidental self-injury (HoNOSCA domain), most participants across all three groups had “no problem of this kind” (67.2% in Group 1, 66.8% in Group 2, and 81.5% in Group 3). The groups differed significantly in the proportion of participants with varying degrees of self-injury and suicidal intent (p < 0.001).

**Table 2:** Severity of mental health problems, impairment and functioning and experiences of the milestone cohort assessed at baseline by medication grouP (T1, N = 690).

	Mean (SD) or N (%)			
	Group 1* (N = 186)	Group 2* (N = 304)	Group 3* (N = 200)	p-value**
**SEVERITY OF MENTAL HEALTH PROBLEMS**				
**Clinician rated severity of psychopathology (CGI-S)**				
*Not at all ill*	15 (8.1%)	18 (5.9%)	29 (14.5%)	**0.018**
*Borderline/mildly/moderately ill*	108 (58.1%)	179 (58.9%)	125 (62.5%)	
*Markedly ill or more severe*	38 (20.4%)	65 (21.4%)	31 (15.5%)	
*Missing*	25 (13.4%)	42 (13.8%)	15 (7.5%)	
**Mental health (HoNOSCA; range 0–52)**	13.9 (9.6)	12.8 (8.6)	9.3 (7.0)	**<0.001**
**Lifetime suicide attempt**				
*Yes*	56 (30.1%)	90 (29.6%)	34 (17.0%)	**<0.001**
*No*	116 (62.4%)	193 (63.5%)	161 (80.5%)	
*Missing*	14 (7.5%)	21 (6.9%)	5 (2.5%)	
**Non-accidental self-injury (HoNOSCA domain)**				
*No problem of this kind*	125 (67.2%)	203 (66.8%)	163 (81.5%)	**<0.001**
*Occasional thoughts about death, or of self-harm not leading to injury. No self-harm or suicidal thoughts*.	21 (11.3%)	25 (8.2%)	15 (7.5%)	
*Non-hazardous self-harm whether or not associated with suicidal thoughts*	9 (4.8%)	20 (6.6%)	12 (6.0%)	
*Moderately severe suicidal intent or moderate non-hazardous self-harm*	16 (8.6%)	28 (9.2%)	7 (3.5%)	
*Serious suicidal attempt or serious deliberate self-injury*	12 (6.5%)	17 (5.6%)	3 (1.5%)	
*Missing*	3 (1.6%)	11 (3.6%)	0 (0.0%)	
**Quality of life (WHOQOL-BREF; range 4–20)**				
*Psychological*	17.2 (3.9)	17.3 (3.7)	18.6 (3.3)	**<0.001**
*Physical*	20.8 (3.7)	20.7 (3.8)	20.9 (3.0)	**0.048**
*Social*	10.4 (2.5)	10.1 (2.3)	10.6 (2.4)	**0.007**
*Environmental*	30.8 (5.1)	29.7 (5.4)	30.1 (4.7)	**<0.001**
*Total*	79.2 (12.1)	77.9 (12.1)	80.2 (10.5)	**<0.001**
**Everyday functional skills (SLOF)**				
*Physical functioning*	24.1 (1.6)	24.2 (1.6)	24.1 (1.5)	**0.023**
*Personal care skills*	33.1 (2.6)	33.5 (2.4)	33.7 (2.7)	**<0.001**
*Interpersonal relationships*	24.6 (6.8)	25.8 (6.2)	27.5 (6.6)	**<0.001**
*Social acceptability*	30.7 (3.9)	31.1 (3.6)	32.3 (3.1)	**<0.001**
*Activities*	48.3 (7.8)	49.9 (5.7)	50.7 (5.7)	**<0.001**
*Work skills*	22.8 (6.3)	23.9 (5.3)	24.1 (6.0)	**<0.001**
*Total*	183.5 (21.7)	188.8 (17.7)	192.5 (17.5)	**<0.001**
**Life events (range 0–13)**	1.7 (1.7)	2.1 (1.7)	1.8 (1.6)	**<0.001**
**Bullying**				
Victim	72 (38.7%)	110 (36.2%)	88 (44.0%)	**<0.001**
Bully/victim	70 (37.6%)	119 (39.1%)	66 (33.0%)	
Bully	4 (2.2%)	13 (4.3%)	5 (2.5%)	
Non-involved	37 (19.9%)	52 (17.1%)	37 (18.5%)	
Missing	3 (1.6%)	10 (3.3%)	4 (2.0%)	

* Group 1 = Continuous Medication, Group 2 = Intermittent Medication, Group 3 = Never Medicated.

** P-values in bold are statistically significant.

There were significant differences in the overall quality of life scores rated with the WHOQOL-BREF (p < 0.001) among the three groups (79.2 for Group1, 77.9 for Group2, and 80.2 for Group3). Significant differences were found among the three groups in any functional skill domain or total SLOF score (p < 0.001), and the same applies to the mean number of life events among the three groups (183.5 for Group1, 188.8 for Group2, and 192.5 for Group3). The proportion of participants involved in bullying incidents differed significantly between the three medication groups (p < 0.001). In summary, Group 3 generally showed better mental health outcomes, with lower psychopathology severity, fewer suicide attempts, and better social quality of life than Groups 1 and 2.

Table 3 presents the distribution of psychotropic prescriptions at baseline and T4, distinguishing between continuously medicated patients and intermittently medicated patients. Most continuously medicated patients received only one medication. Antidepressants were the most common, prescribed to 63.9% of patients at T1 and 58.1% at T4. Second-generation antipsychotics were used by 31.7% at T1 and 32.3% at T4, while benzodiazepine use dropped from 9.1% at baseline to 4.3% at T4. Among intermittently medicated patients, the proportion using each drug class was significantly lower than in continuously medicated individuals. For instance, antidepressant prescriptions fell from 35.9% at T1 to 11.8% at T4, and second-generation antipsychotics dropped from 15.5%–3.6%. The average number of psychotropic medications per patient in this group also decreased from 0.9 at T1 to 0.4 at T4. In the intermittent group, 133 patients (43.7%) received medication at one time point, with 75 patients (24.7%) treated only at T1. Additionally, 89 patients (29.3%) were treated at two timepoints, while 82 patients (27.0%) received treatment at three time points during the observation period.

**Table 3:** Pattern of prescriptions in the whole sample at t1 and t4 (n = 690)*.

	N of patients receiving any drugs from each class among medicated patients (N = 186) (%)	N of patients receiving any drugs from each class among intermittent medicated patients (N = 304) (%)	Mean N of drugs from each class (SD) in the whole sample	N of drugs range in the whole sample
TIME POINT 1				
First-Generation Antipsychotic	8 (4.3%)	8 (2.6%)	2.0 (0.9)	1-3
Second-Generation Antipsychotic**	59 (31.7%)	47 (15.5%)	1.8 (0.8)	1-4
Mood Stabilizers	19 (10.2%)	7 (2.3%)	1.5 (0.7)	1-3
Antidepressants	119 (63.9%)	109 (35.9%)	1.5 (0.7)	1-5
Benzodiazepines	17 (9.1%)	30 (9.9%)	1.5 (0.9)	1-4
Stimulants	60 (32.3%)	44 (14.5%)	1.3 (0.6)	1-3
Mean N of Drugs for Each Patient (SD)	1.5 (0.8)	0.9 (0.8)		
TIME POINT 4				
First-Generation Antipsychotic	12 (6.5%)	2 (0.7%)	1.8 (1.0)	1-3
Second-Generation Antipsychotic**	60 (32.3%)	11 (3.6%)	1.5 (0.7)	1-3
Mood Stabilizers	20 (10.8%)	8 (2.6%)	1.8 (0.8)	1-3
Antidepressants	108 (58.1%)	36 (11.8%)	1.4 (0.8)	1-5
Benzodiazepines	8 (4.3%)	16 (5.3%)	1.6 (0.7)	1-3
Stimulants	60 (32.3%)	10 (3.3%)	1.5 (0.6)	1-3
Mean N Of Drugs for Each Patient (SD)	1.5 (0.8)	0.4 (0.7)		

*The patients listed in specific psychotropic medication categories at baseline (T1) may not be the same patients taking the corresponding prescriptions at follow-up (T4).

** Patients receiving clozapine have been included in this row (at T1 and T4).

Figures S5 and S6 illustrate the number of medications administered at each time point for the continuously and intermittently medicated groups, respectively. The distribution of the number of medications prescribed at each time point, including counts and percentages for both groups, is detailed in Table 5S. Table 6S shows the patterns of medication prescriptions for each class considered at each time point.

The logistic regression model shows that several factors were associated with the risk of severe disorder assessed at the last time point (T4). An improvement in the risk of suicide attempts (p = 0.038), higher everyday functional skills (p = 0.008), higher quality of life (p = 0.001) and being male (p = 0.020) were associated to a reduced risk of severe illness. In addition, continuous medication status (0.051) was marginally associated with a higher risk of severe illness, suggesting that those on continuous medication may face a higher risk. The effects of being a victim of bullying were not found to be statistically significant.

### Course and outcomes of adolescents rated with HoNOSCA

Figure 1 displays the patient- and clinician-rated HoNOSCA ratings across the three medication profiles (e.g., *continuous medication*, *intermittent medication*, and *never medicated*). This analysis revealed associations between patient and clinician assessments across various medication regimens over time. However, there were notable differences in rating magnitudes. In both self-administered and clinician-reported assessments, never-medicated patients showed significantly different ratings compared to patients in the continuous medication group. For the self-administered reports, differences were particularly evident at T3 (p = 0.001) and T4 (p < 0.001). Similarly, for the clinician reports, significant differences were observed at T3 (p = 0.001) and T4 (p < 0.001). Additionally, investigators rate symptom severity were higher than patients’ self-assessments. However, the difference between the self- and clinician-assessed ratings varied significantly across the three psychotropic medication profiles (p < 0.001; for further information, see Fig. S2 in the Supplementary Material).

### Course and outcomes of adolescents rated with ASEBA Battery

**Fig. 2:**
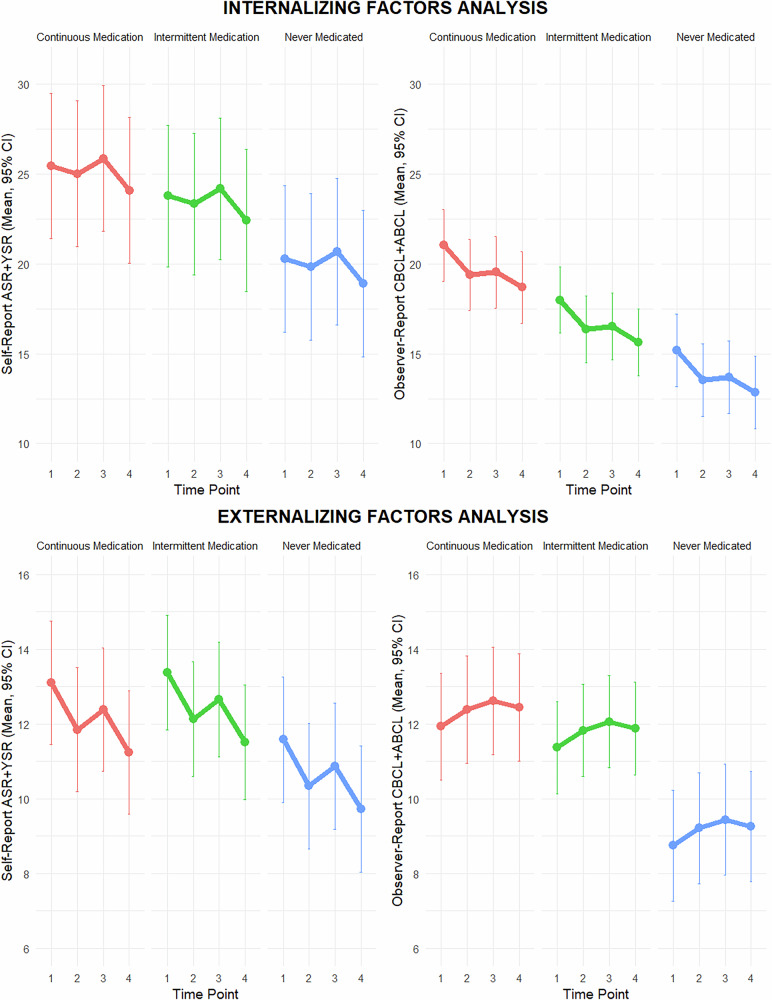
Internalising and externalising domains: self-report (asr + ysr) vs. parent-report (cbcl + abcl) ratings by medication status.

Figure 2 shows the analysis of the internalising and externalising domains based on medication status, comparing self-reported (ASR and YSR scores) with parental reports (CBCL and ABCL scores). Regarding internalising factors, there was a distinct trend: patients who never had never been medicated exhibited the lowest ratings, suggesting fewer internalising symptoms, followed by those with an intermittent medication status. Patients taking continuous medication had the highest ratings, indicating more internalising symptoms. This pattern revealed a significant association between medication status and internalising domains, with statistically significant changes across the three medication profiles and over the four time points (p < 0.001) for both patient and parent ratings. In both self- and parent-reported assessments, never-medicated patients showed significantly different ratings compared to patients in the continuous medication group. For self-reports, significant differences were observed at T1 (p = 0.002), T2 (p = 0.001), T3 (p = 0.016), and T4 (p < 0.001). For parent reports, significant differences were found at T1 (p < 0.001), T2 (p < 0.001), T3 (p < 0.001), and T4 (p < 0.001). However, the difference between self- and parent-assessed ratings did vary significantly across the three psychotropic medication profiles (p = 0.007; for further information, see Fig. S3 in Supplementary Material).

For the externalising domain based on medication status, the data show a clear trend: patients who had never been medicated displayed the lowest scores, indicating fewer externalising symptoms. Those with intermittent medication status had moderately higher scores, and those on continuous medication exhibited the highest scores, suggesting more reported externalising symptoms. This trend underscores that medication status is significantly associated with externalising factors, as measured by both self-report (ASR and YSR) and parent-report (CBCL and ABCL) assessments. Notably, while statistically significant changes across the three medication profiles and the four time points were observed in both self-report (p = 0.027) and parent-report assessments (p < 0.001), the pattern and magnitude of these changes differed, highlighting a discrepancy in the perception of externalising behaviours between adolescents and their caregivers. According to the parents’ ratings, never-medicated patients showed significantly different ratings compared to patients in the continuous medication group, especially at T1 and T4 (p = 0.034 vs p = 0.019, and p = 0.002 vs p = 0.135, respectively). Additionally, the difference between the self-assessed and parent-assessed ratings varied significantly across the medication regimens (p = 0.038; for further information, see Fig. S4 in the Supplementary Material).

### Correlation between self-reported and observer-reported assessments

Table 3S presents the correlation coefficients between the self- and observer-reported assessments across various time points. When patients and clinicians were compared using the HoNOSCA Total Rating, the overall correlation coefficient was 0.589, indicating a moderate level of association. The correlations at each time point were consistent, ranging from 0.508 at T1 to 0.626 at T4. Regarding internalising factors, the correlation between self-reports (ASR + YSR) and parental reports (CBCL + ABCL) was moderate, with an overall correlation coefficient of 0.605. However, there was an increasing trend in the association over time, starting at 0.576 at T1 to 0.635 at T4. For externalising factors, the correlation between self-reports and parental reports was moderate overall (0.545); however, the association decreased over time, with correlations ranging from 0.563 at T1 to 0.511 at T4. All correlation coefficients were statistically significant (p < 0.001), indicating robust relationships between self- and observer-reported assessments across all measures and time points [30].

## Discussion

As part of the broader MILESTONE project, this study offers a nuanced exploration of clinical trajectories and outcomes among adolescents, aged 17-19, with mental disorders undergoing various medication regimens over two years. Using the wealth of MILESTONE study data and by categorising adolescents into continuous, intermittent, and never medicated groups, we created a comprehensive framework for analysing the association between medication patterns and clinical outcomes. Our results suggest a complex relationship between medication regimens and changes in mental health symptoms, as measured by assessment tools such as the HoNOSCA, YSR, ASR, CBCL, and ABCL. Continuous medication was associated with higher symptom severity scores than intermittent or no medication, possibly reflecting either a more severe initial symptomatology requiring sustained pharmacological treatment or a medication-related paradox, where symptoms persist or intensify despite ongoing treatment due to issues such as treatment resistance or side effects [31].

While the existing literature provides valuable insights into the prevalence and correlates of psychotropic medication use among adolescents with mental disorders, several notable gaps remain. The studies conducted by Olfson et al. [32], Altuwairqi et al. [33], Goodwin et al. [34], and Brown et al. [35] have contributed to our understanding of medication prescribing patterns, prevalence trends, sociodemographic characteristics, and their associations with adverse outcomes. However, these studies primarily offer cross-sectional snapshots or systematic reviews and often lack longitudinal perspectives and detailed assessments of clinical profiles. In Italy, Clavenna et al. [36] conducted an epidemiological study to estimate the treatment coverage for mental disorders in paediatric patients. In this survey involving 59,987 young people in the Lombardy Region, 37.1% had attended CAMHS at least once, 2.2% had been hospitalised, and 1.7% had received at least one prescription for psychotropic drugs. Among all users, 23.1% had disorders requiring a high level of care, such as recurrent drug prescriptions and inpatient treatments.

Discrepancies between patient and clinician assessments, particularly instances in which patients reported greater symptom decreases than clinicians, highlight the subjective nature of symptom changes and may indicate clinicians’ cautious perspectives or possible underestimation of patient-reported improvements. Recognising these differences in clinical practice is essential to enhance communication and treatment planning [37–39]. Our findings emphasise the importance of considering psychosocial dimensions in mental health trajectories. Adolescents with mental health conditions navigate through complex social environments that can exacerbate or alleviate their symptoms. Factors such as family dynamics, bullying experiences, and major life events play critical roles and were considered in this study. For example, the prevalence of bullying and its correlation with worse mental health outcomes underscores the need for robust support systems in educational and community settings to mitigate these impacts [40, 41].

### Service transitions and treatment continuity

The transition from CAMHS to AMHS emerged as a critical period, laden with potential disruptions in care continuity. Adolescents undergoing this transition are particularly vulnerable and often experience significant shifts in their support systems and care protocols. Our data reflect this transitional turbulence, with some participants showing unexpected spikes in symptom severity scores at the final assessment points. This phenomenon may be attributed to the challenges of adjusting to new care settings, differences in treatment approaches between paediatric and adult services, or gaps in service provision [42–44].

### Limitations

The MILESTONE cohort study had several limitations. The most important limitation was the potential selection bias, as CAMHS were affiliated with the MILESTONE consortium and were not randomly selected. The missing data analysis showed a potential bias, with more missing parental information in young people with severe diagnosis. A common limitation of studies on medication prescriptions is the lack of adherence data: we gathered details on prescribed medications, recipients, and timing but could not confirm if patients took the medications as prescribed. This does not affect the never medicated group; however, for the intermittently and continuously medicated groups, adherence cannot be verified. Despite this, the distinct clinical profiles and changes observed over time suggest that most patients were likely to adhere to their treatment plans.

## Conclusions

This study examined the association of different medication regimens with the clinical trajectories of adolescents with mental disorders. Through a better understanding of treatment adherence, symptomatology, and critical transition periods, we can develop more effective approaches to support young people facing psychological problems. Longitudinal studies of adolescents undergoing pharmacological treatment face challenges, including ethical considerations and distinguishing treatment effects from natural development. An accurate diagnosis is crucial because the symptoms overlap with typical adolescent behaviours. Further research is needed to understand developmental trajectories, symptom progression, and treatment variations, while we also comply with necessary ethical standards. Our study aimed to fill the gaps in the literature using a unique dataset from a European sample to inform clinical practice and policy decisions regarding adolescent mental health management.

## Supplementary information

Suppelementary Material

## Data Availability

The data that support the findings of this study are available from the corresponding author upon reasonable request.

## References

[1] Blakemore SJ. Adolescence and mental health. Lancet. 2019;393:2030–1. 31106741 10.1016/S0140-6736(19)31013-X

[2] Galván A. Adolescence, brain maturation and mental health. Nat Neurosci. 2017;20:503–4. 28352110 10.1038/nn.4530

[3] MacLeod KB, Brownlie EB. Mental health and transitions from adolescence to emerging adulthood: developmental and diversity considerations. Can J Community Ment Health. 2014;33:77–86.

[4] Blakemore SJ, Mills KL. Is adolescence a sensitive period for sociocultural processing? Annu Rev Psychol. 2014;65:187–207. 24016274 10.1146/annurev-psych-010213-115202

[5] Lin J, Guo W. The research on risk factors for adolescents’ mental health. Behav Sci. 2024;14:263. 38667059 10.3390/bs14040263 PMC11047495

[6] Sacco R, Camilleri N, Eberhardt J, Umla-Runge K, Newbury-Birch D A systematic review and meta-analysis on the prevalence of mental disorders among children and adolescents in Europe. Eur Child Adolesc Psychiatry. 2022;1–18. 10.1007/s00787-022-02131-2 PMC9800241 36581685

[7] Polanczyk GV, Salum GA, Sugaya LS, Caye A, Rohde LA. Annual research review: a meta‐analysis of the worldwide prevalence of mental disorders in children and adolescents. J Child Psychol Psychiatry. 2015;56:345–65. 25649325 10.1111/jcpp.12381

[8] Kovess-Masfety V, Husky MM, Keyes K, Hamilton A, Pez O, Bitfoi A, et al. Comparing the prevalence of mental health problems in children 6-11 across Europe. Soc Psychiatry Psychiatr Epidemiol. 2016;51:1093–103. 27314494 10.1007/s00127-016-1253-0 PMC5279951

[9] Hossain MM, Nesa F, Das J, Aggad R, Tasnim S, Bairwa M, et al. Global burden of mental health problems among children and adolescents during COVID-19 pandemic: An umbrella review. Psychiatry Res. 2022;317:114814. 36055064 10.1016/j.psychres.2022.114814 PMC9420079

[10] Scheiner C, Grashoff J, Kleindienst N, Buerger A. Mental disorders at the beginning of adolescence: prevalence estimates in a sample aged 11-14 years. Public Health Pract. 2022;4:100348. 10.1016/j.puhip.2022.100348 PMC9761382 36545674

[11] Seker S, Boonmann C, d’Huart D, Bürgin D, Schmeck K, Jenkel N, et al. Mental disorders into adulthood among adolescents placed in residential care: A prospective 10-year follow-up study. Eur Psychiatry. 2022;65:e40. 35730184 10.1192/j.eurpsy.2022.30 PMC9280920

[12] Solmi M, Radua J, Olivola M, Croce E, Soardo L, Salazar de Pablo G, et al. Age at onset of mental disorders worldwide: large-scale meta-analysis of 192 epidemiological studies. Mol Psychiatry. 2022;27:281–95. 34079068 10.1038/s41380-021-01161-7 PMC8960395

[13] McGrath JJ, Al-Hamzawi A, Alonso J, Altwaijri Y, Andrade LH, Bromet EJ, et al. Age of onset and cumulative risk of mental disorders: a cross-national analysis of population surveys from 29 countries. Lancet Psychiatry. 2023;10:668–81. 37531964 10.1016/S2215-0366(23)00193-1 PMC10529120

[14] Smogur M, Onesanu A, Plessen KJ, Eap CB, Ansermot N. Psychotropic drug prescription in children and adolescents: approved medications in European countries and the United States. J Child Adolesc Psychopharmacol. 2022;32:80–8. 35138922 10.1089/cap.2021.0027

[15] Kearns GL, Abdel-Rahman SM, Alander SW, Blowey DL, Leeder JS, Kauffman RE. Developmental pharmacology-drug disposition, action, and therapy in infants and children. N Engl J Med. 2003;349:1157–67. 13679531 10.1056/NEJMra035092

[16] Hendrickx G, De Roeck V, Maras A, Dieleman G, Gerritsen S, Purper-Ouakil D, et al. Challenges during the transition from child and adolescent mental health services to adult mental health services. BJPsych Bull. 2020;44:163–8. 31931898 10.1192/bjb.2019.85 PMC8058856

[17] Singh SP, Paul M, Ford T, Kramer T, Weaver T. Transitions of care from child and adolescent mental health services to adult mental health services (TRACK Study): a study of protocols in greater London. BMC Health Serv Res. 2008;8:135. 18573214 10.1186/1472-6963-8-135 PMC2442433

[18] Reneses B, Escudero A, Tur N, Agüera-Ortiz L, Moreno DM, Saiz-Ruiz J, et al. The black hole of the transition process: dropout of care before transition age in adolescents. Eur Child Adolesc Psychiatry. 2023;32:1285–95. 35048161 10.1007/s00787-021-01939-8 PMC10276128

[19] Singh SP, Tuomainen H, Girolamo Gde, Maras A, Santosh P, McNicholas F, et al. Protocol for a cohort study of adolescent mental health service users with a nested cluster randomised controlled trial to assess the clinical and cost-effectiveness of managed transition in improving transitions from child to adult mental health services (the MILESTONE study). BMJ Open. 2017;7:e016055. 29042376 10.1136/bmjopen-2017-016055 PMC5652531

[20] D’Avanzo B, Lovaglio P, Parabiaghi A, Conti P, Frigerio A, Molteni M, et al. Health of the nation outcome scales for children and adolescents (HoNOSCA): psychometric properties of the Italian version. Child Youth Serv Rev. 2018;94:340–6.

[21] Gowers SG, Harrington RC, Whitton A, Beevor A, Lelliott P, Jezzard R, et al. Health of the nation outcome scales for children and adolescents (HoNOSCA). glossary for HoNOSCA score sheet. Br J Psychiatry. 1999;174:428–31. 10616610 10.1192/bjp.174.5.428

[22] Achenbach TM, Rescorla LA Manual for the ASEBA School-Age Forms & Profiles. Burlington, VT: University of Vermont, Research Center for Children, Youth, & Families.; 2001.

[23] Achenbach TM, Rescorla L Manual for the ASEBA Preschool Forms & Profiles. Burlington: University of Vermont, Research Center for Children, Youth, and Families. 2000.

[24] Schneider LC, Struening EL, Elmer Struening TL SLOF: a behavioral rating scale for assessing the mentally ill. 1983. http://swra.oxfordjournals.org/. 10.1093/swra/19.3.9 10264257

[25] Mucci A, Rucci P, Rocca P, Bucci P, Gibertoni D, Merlotti E, et al. The specific level of functioning scale: construct validity, internal consistency and factor structure in a large Italian sample of people with schizophrenia living in the community. Schizophr Res. 2014;159:144–50. 25182540 10.1016/j.schres.2014.07.044

[26] de Girolamo G, Rucci P, Scocco P, Becchi A, Coppa F, D’Addario A, et al. Quality of life assessment: validation of the italian version of the WHOQOL-Brief. Epidemiol Psichiatr Soc. 2000;9:45–55. 10859875 10.1017/s1121189x00007740

[27] Wolke D, Sapouna M. Big men feeling small: childhood bullying experience, muscle dysmorphia and other mental health problems in bodybuilders. Psychol Sport Exerc. 2008;9:595–604.

[28] Busner J, Targum SD. The clinical global impressions scale: applying a research tool in clinical practice. Psychiatry. 2007;4:28–37. 20526405 PMC2880930

[29] Van Buuren S, Groothuis-Oudshoorn K. mice: Multivariate imputation by chained equations in R. Journal of statistical software. 2011;45:1–67.

[30] Singh SP, Tuomainen H, Bouliotis G, Canaway A, De Girolamo G, Dieleman GC, et al. Effect of managed transition on mental health outcomes for young people at the child-adult mental health service boundary: a randomised clinical trial. Psychol Med. 2023;53:2193–204. 37310306 10.1017/S0033291721003901 PMC10123823

[31] Smith SW, Hauben M, Aronson JK. Paradoxical and bidirectional drug effects. Drug Saf. 2012;35:173–89. 22272687 10.2165/11597710-000000000-00000

[32] Olfson M, He JP, Merikangas KR. Psychotropic medication treatment of adolescents: results from the national comorbidity survey-adolescent supplement. J Am Acad Child Adolesc Psychiatry. 2013;52:378–88. 23582869 10.1016/j.jaac.2012.12.006 PMC3664537

[33] Altuwairqi Y. Trends and prevalence of psychotropic medication use in children and adolescents in the period between 2013 and 2023: a systematic review. Cureus. 2024;16:e55452. 38571846 10.7759/cureus.55452 PMC10987897

[34] Goodwin R, Gould MS, Blanco C, Olfson M. Prescription of psychotropic medications to youths in office-based practice. Psychiatr Serv. 2001;52:1081–7. 11474055 10.1176/appi.ps.52.8.1081

[35] Brown TL, Gutierrez PM, Grunwald GK, DiGuiseppi C, Valuck RJ, Anderson HD. Access to psychotropic medication via prescription is associated with choice of psychotropic medication as suicide method: a retrospective study of 27,876 suicide attempts. J Clin Psychiatry. 2018;79:16348. 10.4088/JCP.17m11982 30418710

[36] Clavenna A, Cartabia M, Sequi M, Costantino MA, Bortolotti A, Fortino I, et al. Burden of psychiatric disorders in the pediatric population. Eur Neuropsychopharmacol. 2013;23:98–106. 22561004 10.1016/j.euroneuro.2012.04.008

[37] Beharie N, Scheidell JD, Quinn K, McGorray S, Vaddiparti K, Kumar PC, et al. Associations of adolescent exposure to severe violence with substance use from adolescence into adulthood: direct versus indirect exposures. Subst Use Misuse. 2019;54:191–202. 30541369 10.1080/10826084.2018.1495737 PMC6482818

[38] Nilsen W, Karevold EB, Kaasbøll J, Kjeldsen A. Nuancing the role of social skills- a longitudinal study of early maternal psychological distress and adolescent depressive symptoms. BMC Pediatr. 2018;18:133. 29636005 10.1186/s12887-018-1100-4 PMC5891909

[39] Laurenssen EMP, Hutsebaut J, Feenstra DJ, Van Busschbach JJ, Luyten P. Diagnosis of personality disorders in adolescents: a study among psychologists. Child Adolesc Psychiatry Ment Health. 2013;7:3. 23398887 10.1186/1753-2000-7-3 PMC3583803

[40] Herrenkohl TI, Kosterman R, Hawkins JD, Mason WA. Effects of growth in family conflict in adolescence on adult depressive symptoms: mediating and moderating effects of stress and school bonding. J Adolesc Health. 2009;44:146–52. 19167663 10.1016/j.jadohealth.2008.07.005 PMC2666128

[41] Kehusmaa J, Ruotsalainen H, Männikkö N, Alakokkare AE, Niemelä M, Jääskeläinen E, et al. The association between the social environment of childhood and adolescence and depression in young adulthood - a prospective cohort study. J Affect Disord. 2022;305:37–46. 35231482 10.1016/j.jad.2022.02.067

[42] Singh SP, Paul M, Ford T, Kramer T, Weaver T, McLaren S, et al. Process, outcome and experience of transition from child to adult mental healthcare: multiperspective study. Br J Psychiatry. 2010;197:305–12. 20884954 10.1192/bjp.bp.109.075135

[43] Ragnhildstveit A, Tuteja N, Seli P, Smart L, Uzun N, Bass LC, et al. Transitions from child and adolescent to adult mental health services for eating disorders: an in-depth systematic review and development of a transition framework. J Eat Disord. 2024;12:36. 38454528 10.1186/s40337-024-00984-3 PMC10921655

[44] Hill A, Wilde S, Tickle A. Review: transition from child and adolescent mental health services (CAMHS) to adult mental health services (AMHS): a meta-synthesis of parental and professional perspectives. Child Adolesc Ment Health. 2019;24:295–306. 32677352 10.1111/camh.12339

