# Supplementary material for: Mental health trajectories of adolescents treated with psychotropic medications: insights from the european milestone study
Source: Mol Psychiatry. 2025 Oct 17;31(1):94–103. doi: 10.1038/s41380-025-03307-3 (PMC12700807; doi:10.1038/s41380-025-03307-3)

Molecular Psychiatry

MENTAL HEALTH TRAJECTORIES OF ADOLESCENTS TREATED WITH PSYCHOTROPIC MEDICATIONS: INSIGHTS FROM THE EUROPEAN MILESTONE STUDY

Marta Magno[1#], Donato Martella[1#], Silvia Leone[1], Giovanni Allibrio[2], Angelo Bertani[3], Elisa Caselani[1], Patrizia Conti[4], Samuele Cortese[5], Gwen Dieleman[6], Tomislav Franic[7], Suzanne Gerritsen[6], Deborah Maffezzoni[1], Francesco Margari[8], Ottaviano Martinelli[9], Fiona McNicholas[10,11,12], Rocco Micciolo[13], Renata Nacinovich[14,15], Diane Purper Ouakil[16,17], Adriana Pastore[18], Francesco Rinaldi[19], Paramala Santosh[20], Paolo Scocco[21], Ulrike Schulze[22,23], Swaran Singh+[24], Antonella Squarcia[25], Paolo Stagi[26], Cathy Street[24], Elena Toffol[1], Helena Tuomainen[24], Larissa S van Bodegom[6], Stefano Vicari[27] & Giovanni de Girolamo[1] and on behalf the MILESTONE Consortium*

# Marta Magno and Donato Martella equally contributed to this work

+ Corresponding author:

Prof. Swaran P Singh,

Warwick Medical School, University of Warwick, United Kingdom

E-mail: S.P.Singh@warwick.ac.uk;

* A list of authors and their affiliations appears at the end of the paper

SUPPLEMENTARY MATERIAL

TABLE 1S.

SOCIODEMOGRAPHIC AND CLINICAL CHARACTERISTICS OF THE EXCLUDED SAMPLE (N = 314)

To evaluate the representativeness of the analytical sample (N = 690), we compared participants included in the analysis with those excluded due to missing medication data (N = 314). Overall, the two groups were generally comparable regarding demographic and clinical variables.

Demographic characteristics, such as age and ethnicity, were similar between the groups, with mean ages of 17.7 years for the included group and 17.4 years for the excluded group. Both samples predominantly consisted of individuals from a Caucasian background. However, the gender distribution differed slightly; the included group had a higher proportion of females (64.1%) compared to the excluded group (51.3%).

Clinical severity, as assessed by clinicians, was comparable across levels of illness severity. Mean scores for mental health symptoms, quality of life, and everyday functioning were also similar between the groups. Total HoNOSCA scores among the included groups ranged from 9.3 to 13.9, while the excluded group had a mean score of 13.8. The WHOQOL-BREF total scores for the included sample varied from 77.9 to 80.2, compared to a mean of 78.7 in the excluded group. Similarly, SLOF total scores for the included participants ranged from 183.5 to 192.5, whereas the excluded group had a mean score of 183.5.

The rates of lifetime suicide attempts and non-accidental self-injury did not significantly differ between groups, although the excluded group had slightly higher proportions of missing data for some clinical variables.

In conclusion, despite minor differences in gender distribution and rates of missing data, the included sample appears to be broadly representative of the full cohort.

	**N (%) or mean (SD)**
**Gender**	
Female	161 (51.3%)
Male	153 (48.7%)
**Age**	17.4 (0.31)
**Ethnicity**	
Caucasian	239 (76.1%)
Other ethnic groups	34 (10.8%)
Missing	41 (13.1%)
**Living situation**	
With biological parents	113 (36.0%)
With one biological parent	70 (22.3%)
Adoptive/foster parent(s) or other living arrangements	85 (27.1%)
Missing	46 (14.6%)
**Current education**	
Secondary/vocational	4 (1.3%)
Higher (under/postgraduate)	15 (4.8%)
No current school attendance	170 (54.1%)
Other	7 (2.2%)
Missing	118 (37.6%)
**Clinician rated severity of psychopathology (CGI-S)**	
Not at all ill	27 (8.6%)

Borderline/mildly/moderately ill	164 (52.2%)
Markedly ill or more severe	65 (20.7%)
Missing	58 (18.5%)
**Mental health (HoNOSCA; range 0–52)**	13.8 (10.3)
**Lifetime suicide attempt**	
Yes	83 (24.6%)
No	178 (56.7%)
Missing	53 (16.9%)
**Non-accidental self-injury (HoNOSCA domain)**	
No problem of this kind	167 (53.2%)
Occasional thoughts about death, or of self-harm not leading to injury. No self-harm or suicidal thoughts.	23 (7.3%)
Non-hazardous self-harm whether or not associated with suicidal thoughts	25 (8.0%)
Moderately severe suicidal intent or moderate non-hazardous self-harm	19 (6.1%)
Serious suicidal attempt or serious deliberate self-injury	12 (3.8%)
Missing	68 (21.6%)
**Quality of life (WHOQOL-BREF; range 4–20)**	
Psychological	18.2 (3.8)
Physical	20.7 (3.7)
Social	10.3 (2.7)
Environmental	29.5 (5.4)
Total	78.7 (12.8)
**Everyday functional skills (SLOF)**	
Physical functioning	24.1 (1.4)
Personal care skills	32.6 (3.7)
Interpersonal relationships	25.8 (7.0)
Social acceptability	30.9 (3.5)
Activities	47.9 (8.7)
Work skills	22.1 (6.5%)
Total	183.5 (22.9)
**Life events (range 0–13)**	1.8 (1.6)
**Bullying**	
Victim	72 (22.9%)
Bully/victim	92 (29.3%)
Bully	17 (5.4%)
Non-involved	38 (12.1%)
Missing	95 (30.3%)

Table 2S.

LIST OF STANDARDISED TOOLS USED

TOOLS	DESCRIPTION	RANGE	ADMINISTERED TO
Achebanch System of Empirically Based Assessment (ASEBA)	The Achenbach System of Empirically-Based Assessment [ASEBA] have been used extensively in different contexts to evaluate emotional and behavioural problems in the last 6 months.	Higher scores indicate more emotional/behavioural problems	Patients and parents
Clinical Global Impression – Severity scale (CGI-S)	Instrument to measure the severity of the disorder	1-7	Clinicians
Health of the Nation Outcome Scale for Children and Adolescents (HoNOSCA)	The Health of the Nation Outcome Scale for Children and Adolescents [HoNOSCA] measures general health and social functioning in child and adolescent attending mental health services	0-52	Patients, parents and clinicians
World Health Organization Quality of Life Brief Inventory (WHOQOL-BREF)	Self-administered questionnaire to assess subjective health and well-being over the previous two weeks. It covers four domains: physical health, psychological health, social relationships, and environment.	0-100	Patients
Specific Levels of Functioning (SLOF)	To assess psychosocial functioning in six subscales: physical functioning, self-care skills, interpersonal relationships, social acceptance, community life activities, and work skills.	43-125	Parents
Life events	Instrument developed specifically for this project to assess life events	0-13	Patients
Retrospective Bullying and Friendship Interview Schedule (adapted version)	Instrument used to assess the patient experiences with bullying in different settings	NA	Patients

TABLE 3S.

CORRELATION BETWEEN SELF-REPORTED AND OBSERVER-REPORTED ASSESSMENTS

	Correlation Coefficient	CI (95%)	p-value
**HoNOSCA Total Rating - Patients vs. Clinicians**			
**Time Point 1**	0.508	0.451 – 0.561	< 0.001
**Time Point 2**	0.608	0.559 – 0.653	< 0.001
**Time Point 3**	0.599	0.549 – 0.645	< 0.001
**Time Point 4**	0.626	0.578 – 0.669	< 0.001
**Overall**	0.589	0.564 – 0.613	< 0.001
**Internalising Factors: Self-Report (ASR+YSR) vs. Parent-report (CBCL+ABCL)**			
**Time Point 1**	0.576	0.523 -0.623	< 0.001
**Time Point 2**	0.595	0.544 – 0.641	< 0.001
**Time Point 3**	0.616	0.567 – 0.660	< 0.001
**Time Point 4**	0.635	0.588 – 0.677	< 0.001
**Overall**	0.605	0.581 – 0.628	< 0.001
**Externalising Factors: Self-Report (ASR+YSR) vs. Parent-report (CBCL+ABCL)**			
**Time Point 1**	0.563	0.509 – 0.612	< 0.001
**Time Point 2**	0.535	0.479 – 0.587	< 0.001
**Time Point 3**	0.586	0.534 – 0.633	< 0.001
**Time Point 4**	0.511	0.453 – 0.564	< 0.001
**Overall**	0.545	0.519 – 0.571	< 0.001

TABLE 4S.

DISTRIBUTION OF DIAGNOSES ACROSS MEDICATION GROUPS AT BASELINE

	Group 1* (N = 186)	Group 2* (N = 304)	Group 3* (N = 200)	Total sample (N = 690)
**Anxiety**	28 (15.1%)	38 (12.5%)	27 (13.5%)	93 (13.4%)
**Depression**	42 (22.6%)	62 (20.4%)	29 (14.5%)	133 (19.3%)
**Neurodevelopmental**	69 (37.1%)	85 (28.0%)	75 (37.5%)	229 (33.2%)
**OCD/ED**	19 (10.2%)	34 (11.2%)	23 (11.5%)	76 (11.0%)
**Other**	24 (12.8%)	63 (20.7%)	37 (18.5%)	124 (18.0%)
**Missing**	4 (2.2%)	22 (7.2%)	9 (4.5%)	35 (5.1%)

* Group 1 = Continuous Medication, Group 2 = Intermittent Medication, Group 3 = Never Medicated

The diagnostic category 'Other' comprises conditions including personality disorders, conduct disorders, substance use disorders and schizophrenia

TABLE 5S.

NUMBER OF MEDICATIONS TAKEN AT EACH TIME POINT IN MEDICATED PATIENTS

Medication (n.)	T1	T2	T3	T4
Continuous Medication (N = 186)				
**1**	107 (57.3%)	106 (57.0%)	95 (51.1%)	111 (59.7%)
**2**	60 (32.3%)	57 (30.6%)	67 (36.0%)	57 (30.6%)
**3**	14 (7.5%)	18 (9.7%)	14 (7.5%)	16 (8.6%)
**4**	4 (2.2%)	3 (1.6%)	8 (4.3%)	0 (0.0%)
**5**	1 (0.5%)	2 (1.1%)	2 (1.1%)	2 (1.1%)
Intermittent Medication (N = 304)				
**0**	99 (32.6%)	174 (57.2%)	185 (60.9%)	201 (66.1%)
**1**	159 (52.3%)	99 (32.6%)	100 (32.9%)	79 (26.0%)
**2**	37 (12.1%)	19 (6.2%)	15 (4.8%)	17 (5.6%)
**3**	7 (2.3%)	8 (2.7%)	2 (0.7%)	6 (2.0%)
**4**	2 (0.7%)	3 (1.0%)	2 (0.7%)	0 (0.0%)
**5**	0 (0.0%)	1 (0.3%)	0 (0.0%)	1 (0.3%)

TABLE 6S.

PATTERN OF PRESCRIPTIONS IN THE MEDICATED SAMPLE AT EACH TIME POINT

	T1	T2	T3	T4
Continuous Medication (N = 186)				
**First-Generation Antipsychotic**	8 (4.3%)	8 (4.3%)	9 (4.8%)	12 (6.5%)
**Second-Generation Antipsychotic****	59 (31.7%)	61 (32.8%)	65 (34.9%)	60 (32.3%)
**Mood Stabilizers**	19 (10.2%)	20 (10.8%)	20 (10.8%)	20 (10.8%)
**Antidepressants**	119 (63.9%)	112 (60.2%)	123 (66.1%)	108 (58.1%)
**Benzodiazepines**	17 (9.1%)	18 (9.7%)	15 (8.1%)	8 (4.3%)
**Stimulants**	60 (32.3%)	63 (33.9%)	62 (33.3%)	60 (32.3%)
Intermittent Medication (N = 304)				
**First-Generation Antipsychotic**	8 (2.6%)	3 (1.0%)	3 (1.0%)	2 (0.7%)
**Second-Generation Antipsychotic****	47 (15.5%)	25 (8.2%)	20 (6.6%)	11 (3.6%)
**Mood Stabilizers**	7 (2.3%)	4 (1.3%)	6 (2.0%)	8 (2.6%)
**Antidepressants**	109 (35.9%)	72 (23.7%)	46 (15.1%)	36 (11.8%)
**Benzodiazepines**	30 (9.9%)	10 (3.3%)	12 (3.9%)	16 (5.3%)
**Stimulants**	44 (14.5%)	35 (11.5%)	19 (6.2%)	10 (3.3%)

FIGURE S1

FLOWCHART OF THE STUDY

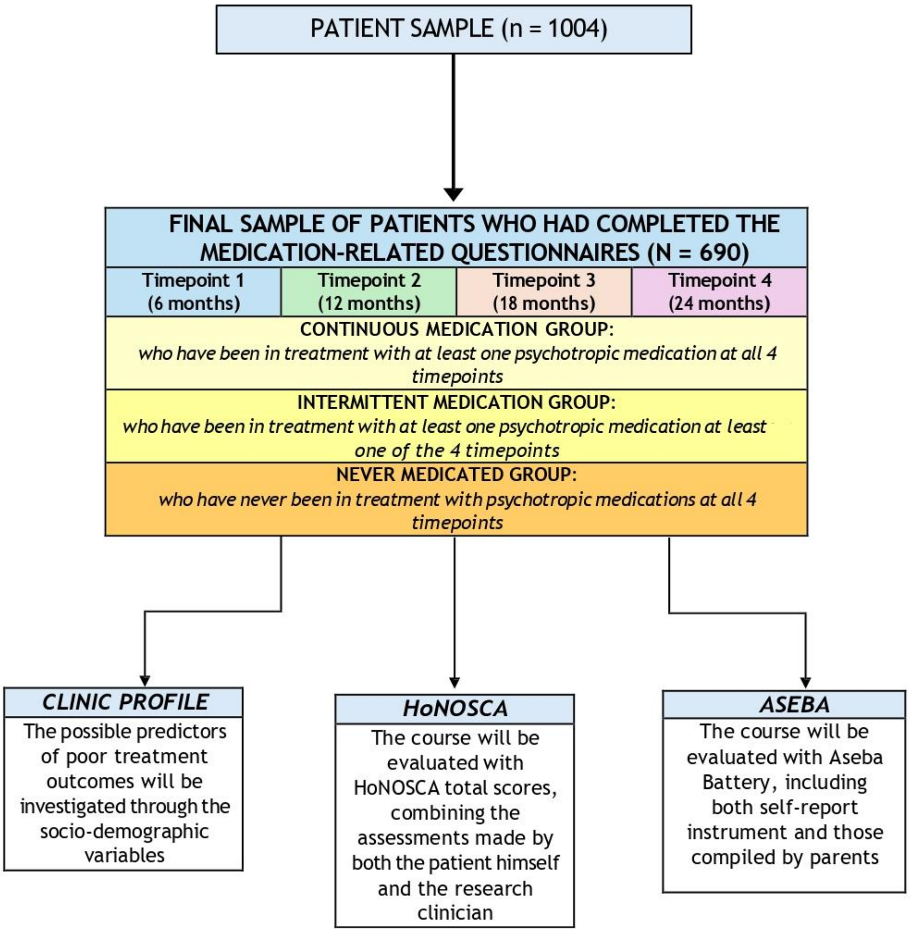

FIGURE S2.

HONOSCA TOTAL RATING - PATIENTS VS. CLINICIANS

Figure S2 provides a general view of patients' courses over 2 years, and shows the HoNOSCA total ratings, comparing patient and clinician ratings across four time points (T1 to T4). Both groups show a general decline in ratings, indicating an overall reduction in symptom severity over time. However, the pattern of decrease differs between the two. Patients reported a more marked improvement in symptoms, particularly noticeable between T1 and T4 ($p < 0.001$), with a significant overall reduction ($p < 0.001$). In contrast, clinicians reported higher severity at T1 compared to patient self-reports and noted a gradual, linear, and significant ($p < 0.001$) decrease in symptoms, which was less pronounced than that reported by patients. For these trends, there is statistically significant difference between the two ratings at any of the four time points ($p = 0.042$).

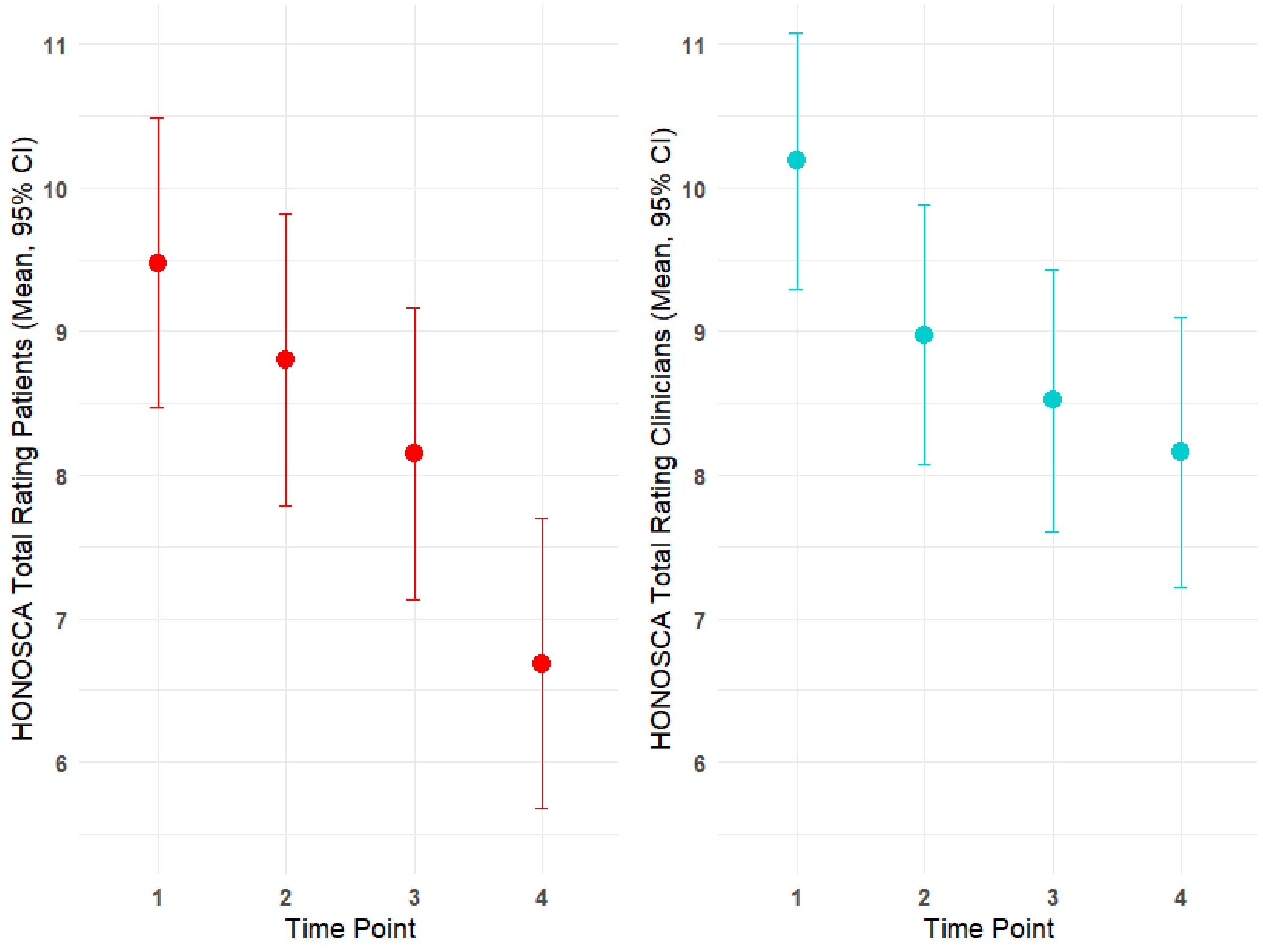

FIGURE S3.

INTERNALISING FACTORS ANALYSIS: SELF-REPORT (ASR+YSR) VS. PARENT-REPORT (CBCL+ABCL)

Figure S3 presents the analysis of ASEBA internalising scales, comparing self-reported (YSR and ASR ratings) and parent-reported (CBCL and ABCL scores) assessments over four time points. There was a significant decrease in ASR and YSR ratings, suggesting an improvement in internalising domains as reported by the subjects themselves ($p < 0.001$). The downward trend observed in the CBCL and ABCL ratings, while indicating a reduction, was statistically significant ($p < 0.001$). For these trends, there was a statistically significant difference between the self-report and parent-report scales at all four time points ($p = 0.017$), highlighting a disparity in perceived internalising symptoms between the adolescents and parent/carers.

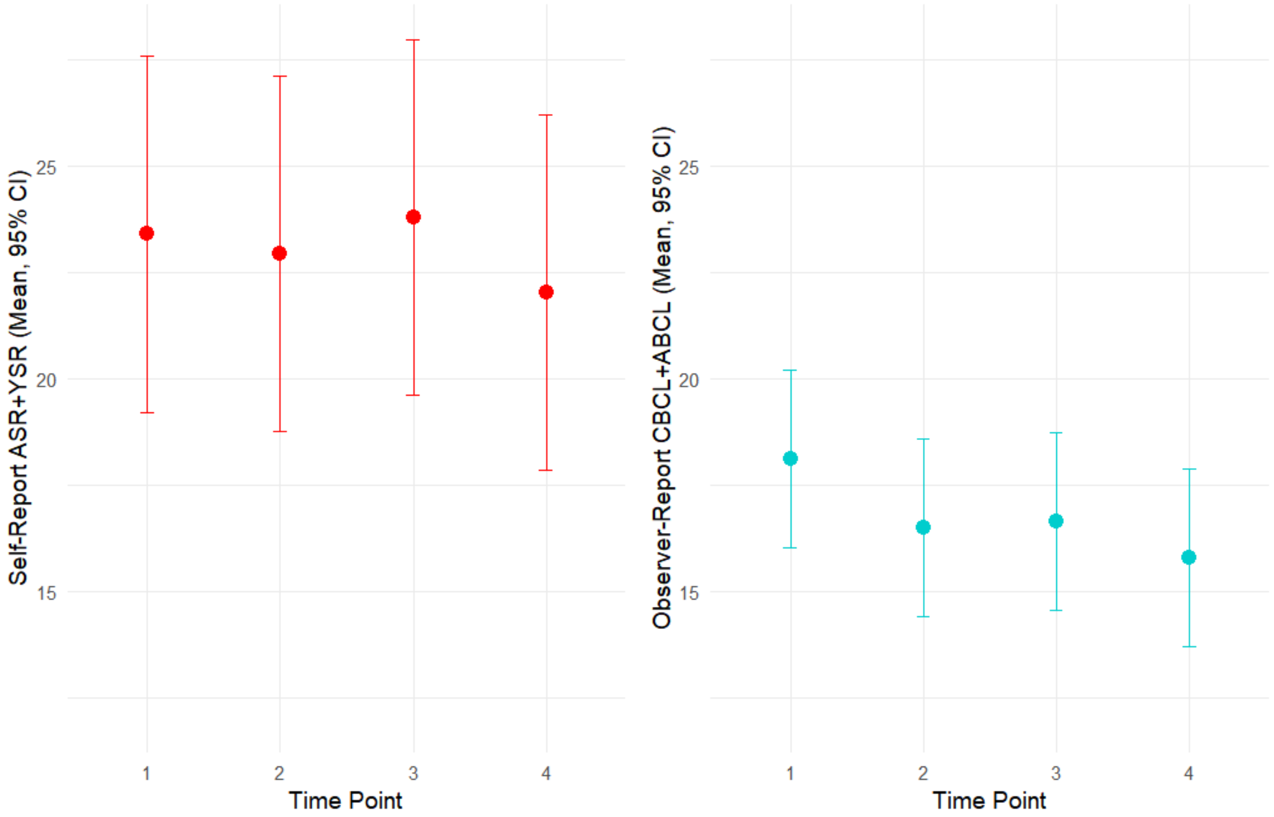

FIGURE S4.

EXTERNALISING FACTORS ANALYSIS: SELF-REPORT (ASR+YSR) VS. PARENT-REPORT (CBCL+ABCL)

Figure S4 illustrates the analysis of ASEBA externalising scales, comparing self-reported (YSR and ASR ratings) and parent-reported (CBCL and ABCL scores) assessments. The results showed a noticeable decrease in the ASR and YSR ratings over time, with average scores significantly diminishing ($p < 0.001$), suggesting a potential improvement in externalising behaviours as self-reported by the subjects. In contrast, the CBCL and ABCL ratings, which are based on parents'/carers' reports, did not exhibit a significant change over time ($p = 0.157$). Additionally, when comparing the changes over time between the self-reports and parents'/carers' reports, there was statistically significant difference between the two scales at any of the time points ($p < 0.001$).

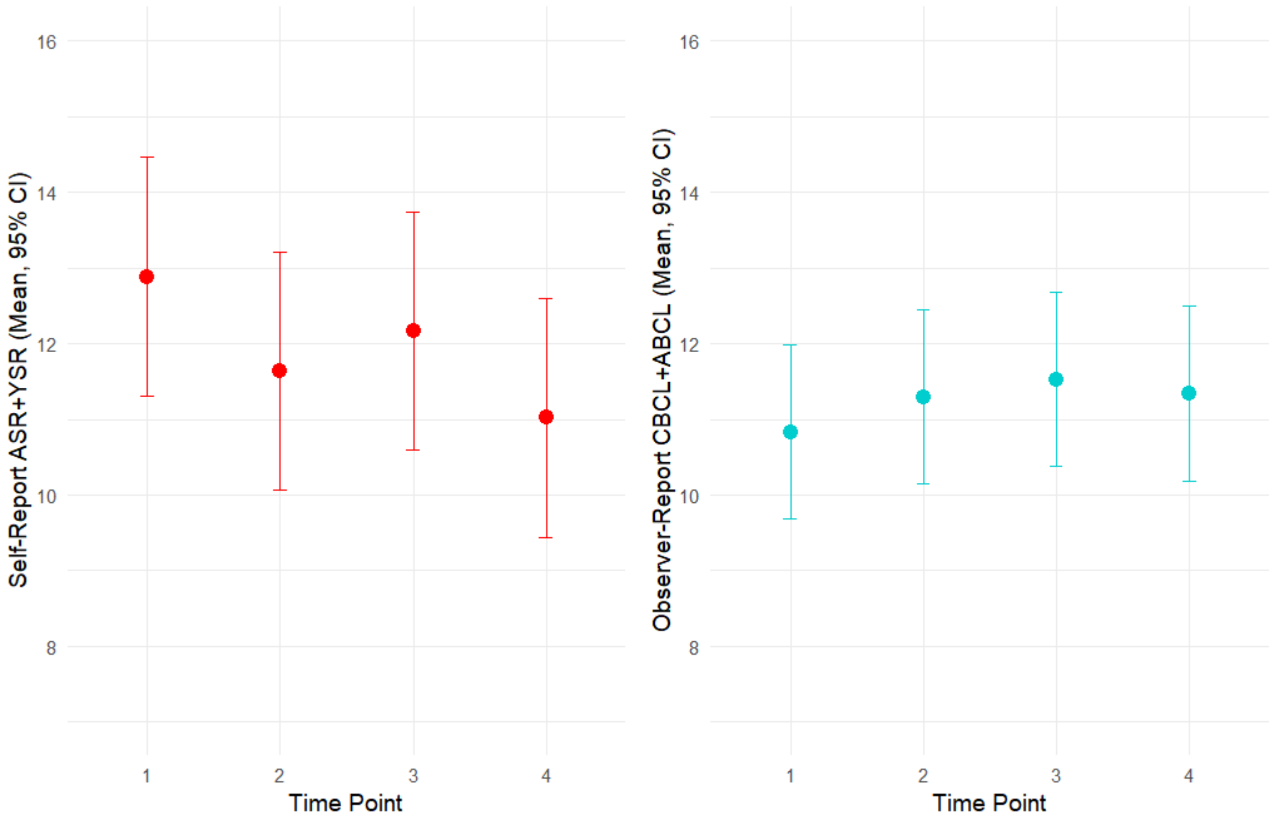

FIGURE S5

NUMBER OF MEDICATIONS TAKEN AT EACH TIME POINT IN PATIENTS WITH CONTINUOUS MEDICATION ADHERENCE (N = 186)

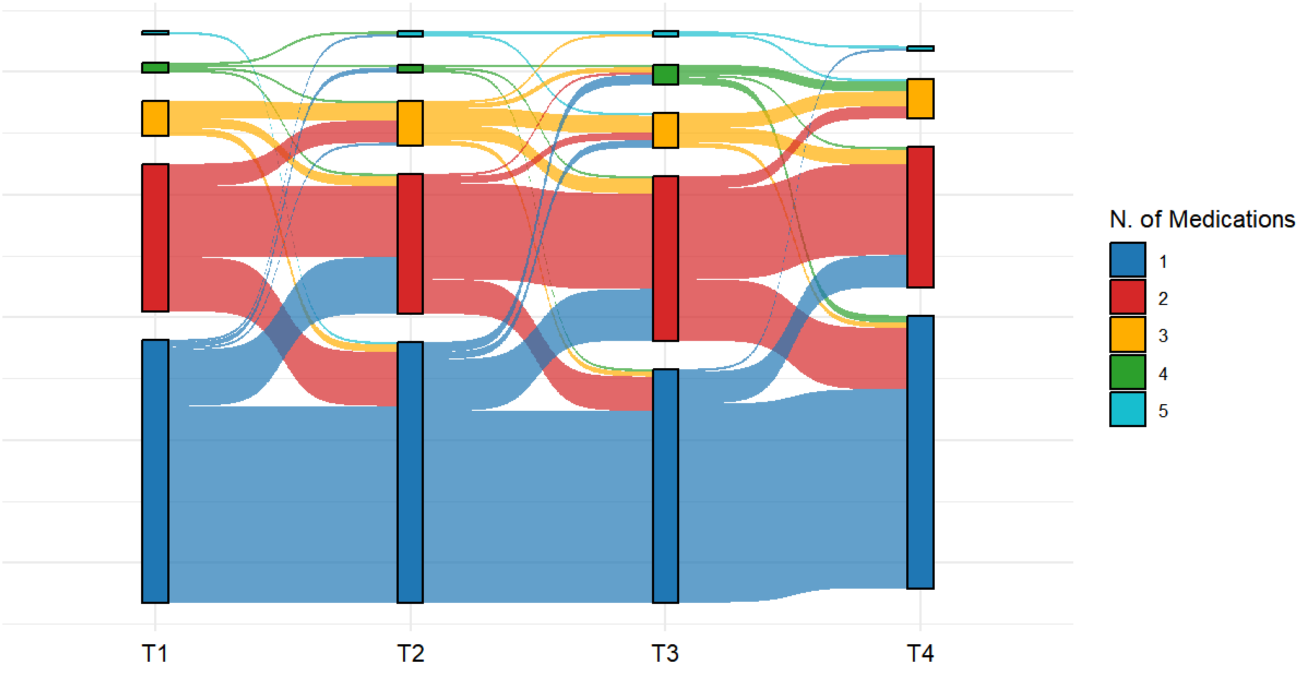

FIGURE S6

NUMBER OF MEDICATIONS TAKEN AT EACH TIME POINT IN PATIENTS WITH INTERMITTENT MEDICATION ADHERENCE (N = 304)

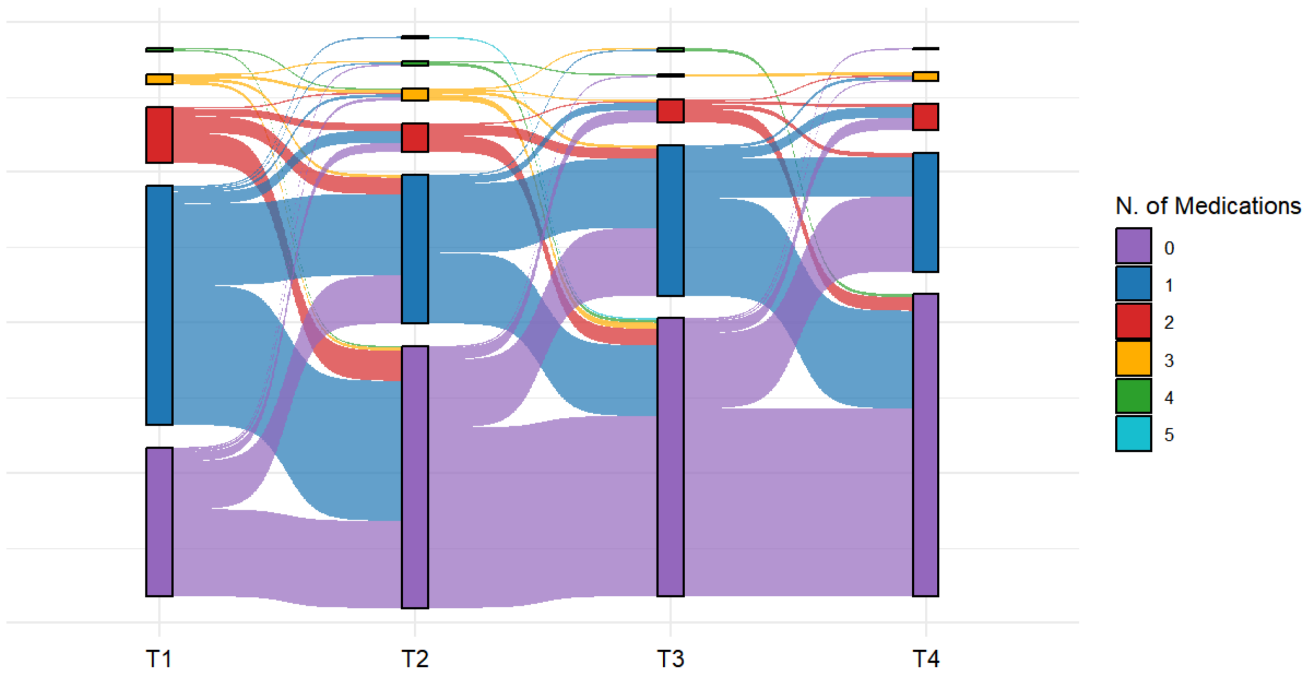

Supplement: Supplementary file 1 — Suppelementary Material [file 41380_2025_3307_MOESM1_ESM.pdf]
